# A Comparative Evaluation of 2.0mm Two-Dimensional Miniplates Versus 2.0mm Three-Dimensional Miniplates in Mandibular Fractures

**DOI:** 10.7759/cureus.21325

**Published:** 2022-01-17

**Authors:** Godvine Sarepally, Swetcha Seethamsetty, Tanveer Karpe, Fazil A Nasyam, Umayra Fatima, Raia Fatema

**Affiliations:** 1 Department of Oral and Maxillofacial Surgery, Dr. Godvine's Clinique, Hyderabad, IND; 2 Department of Oral and Maxillofacial Surgery, Panineeya Mahavidyalaya Institute of Dental Sciences, Hyderabad, IND; 3 Department of Oral and Maxillofacial and Diagnostic Science, Taif University, Taif, SAU; 4 Department of Oral & Maxillofacial Surgery & Diagnostic Sciences, College of Dentistry, Prince Sattam Bin Abdulaziz University, Alkharj, SAU; 5 Dental Department, Deccan College of Medical Sciences, Princess Esra Hospital, Hyderabad, IND; 6 Dentistry, Compass Health Network, Saint Charles, USA

**Keywords:** wound dehiscence, symphysis fracture, paraesthesia, para-symphysis fracture, miniplates

## Abstract

Background: In the past few decades, there has been an increasing interest in obtaining a more instantaneous return to normal function using diverse methods of direct fixation.

Aims and Objectives: To compare the conventional 2-mm 2D (two-dimensional) miniplates and 2-mm 3D (three-dimensional) miniplates in terms of treatment outcome, stability, duration of surgery, and complications of treatment of symphysis and parasymphysis mandibular fractures.

Materials and Methods: 16 patients with clinical and radiological evidence of fractures of the mandible in symphysis and parasymphysis areas treated by open reduction and internal fixation with 2D miniplates and 3D miniplates. The patients were followed up for three months and assessed clinically and radiographically by taking orthopantomograms. The assessment was made on the immediate postoperative day, third day, fifth day, the seventh day, two weeks, three weeks, four weeks, two months, and three months.

Results: Mean intraoperative time taken for 2D miniplate was 54.8 min and for 3D miniplate was 40.6 min. Mild paraesthesia at the soft tissue region supplied by mental nerve was noticed in two patients (25%) of group I, whereas there was no such paraesthesia observed in group II patients. Wound dehiscence and infection were noticed in one patient in group I.

Conclusion: 3D plates seem to be better than conventional 2-mm miniplates for symphysis and parasymphysis fractures.

## Introduction

The main goal in the treatment of any fracture is to predictably restore pre-injury anatomical form with associated aesthetics and function. The concept of bone plating has changed over time, with the introduction of various modifications. Sequentially, bone plates such as compression plates, dynamic compression plates, eccentric, dynamic compression plates, miniplates, and microplates have been introduced, but miniplates are the ones most commonly used [1-3].

There are two fundamentally different philosophies for the treatment of mandible fracture using plates and screws: rigid fixation using compression plates, semi-rigid fixation using miniaturized malleable plates. Luhr felt that mini plates did not offer adequate stabilization of the fractures, thereby necessitating the need for further inter-maxillary fixation [4]. Farmand and Dupoirieux presented 3D (three-dimensional) plates with quadrangular shapes. Easy use, good resistance against torque forces, and compact form of the plates were some of their advantages [3-5].

The purpose of this prospective clinical study was to compare the conventional 2-mm miniplates and 2-mm 3D miniplates in terms of treatment outcome, stability, duration of surgery, and complications of treatment of symphysis and parasymphysis mandibular fractures.

## Materials and methods

The study was approved by the Institutional Ethical Committee with the number "ECR/300/Inst/AP/2014/RR-5". Cases of isolated fractures of mandibular symphysis and parasymphysis were selected for open reduction and internal fixation. This study was conducted on 16 patients with clinical and radiological evidence of fractures of the mandible in symphysis and parasymphysis areas treated by open reduction and internal fixation with 2D (two-dimensional) miniplates and 3D miniplates.

Inclusion criteria of the study are the patients aged between 18-50 years with symphysis and parasymphysis fractures with satisfactory general health conditions without any systemic disease. The patients with co-morbidities, comminuted fractures of the mandible, and malunited or infected fractures were excluded from the study.

A thorough general and medical history of the patients was obtained, and clinical examination was performed with particular reference to the site of the fracture, occlusion, tooth/teeth in the fracture line, ecchymosis/lacerations/abrasions, mouth opening, presence of infection, any neurological disturbances and routine blood and urea examinations were done to rule out any systemic disease.

**Figure 1:**
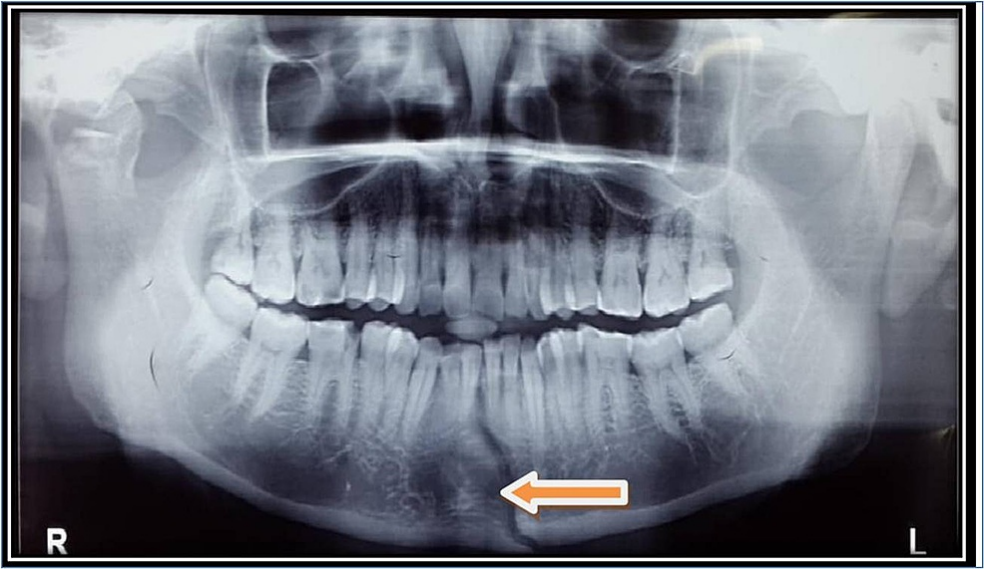
OPG showing fracture site OPG: orthopantomograph

Essential radiographs were taken to correlate the clinical findings, orthopantomograph (OPG), occlusal views of the mandible, intraoral periapical radiographs for evaluating the status of teeth adjacent to the fracture line (Figure 1).

Preliminary treatment in the form of interdental tie wires was placed to temporarily immobilize the fracture fragments. Upper and lower Erich arch bars were placed. Pre-operative antibiotics and analgesics were started. Capsule Amoxycillin 500mg (eight hours), tablet metronidazole 400mg (eight hours), tablet diclofenac sodium 50mg thrice daily, capsule B-complex once daily were prescribed.

Group 1: eight patients received 2D mini plates, one four-hole with gap miniplate at the inferior border of the mandible and another four-hole miniplate at the sub-apical position of the teeth.

Group 2: eight patients received a 3D miniplate with upper crossbar placed in a subapical position of teeth, and injury to dental roots was avoided using mono-cortical screws.

After ruling out head injury and cervical spine injury in the patients and ensuring their complete stabilization, surgery was undertaken. All patients were treated under local anesthesia (injection: lignocaine 2%) with hospitalization. All cases in both groups were treated through an intraoral approach. Erich’s arch bars were placed in the upper and lower arches one day before the surgery in both groups for intra-operative inter-maxillary fixation. And inter-maxillary fixation (IMF) was done before plating to achieve an anatomical reduction of the fracture fragments.

Surgical procedure

After adequate local anesthesia with a nerve block, the area of the incision is infiltrated with local anesthetic solution and vasoconstrictors. In both groups, an intra-oral incision was given in the lower vestibule, 4-5mm below the level of mucogingival junction till the alveolar mucosa. The muscle was incised, retracted, and the fracture site exposed. The length of the incision was made such that it provided adequate exposure to the fracture site.

Adaptation and fixation of the plate

The fractured segments in group I were fixed using 2D 2-mm, four-hole with gap miniplates and using 2.0mm × 10mm monocortical stainless steel screws. The upper plate was a four-hole with a gap plate, fixed so the two-hole lay on each side of the fracture line. The lower plate is also a four-hole plate, fixed so that two holes lay on each side of the fracture line. First, the inferior plate was placed, and later another sub-apical plate was placed 4-5mm above the inferior plate. Postoperative intermaxillary fixation was avoided and done only when occlusion was deranged.

**Figure 2:**
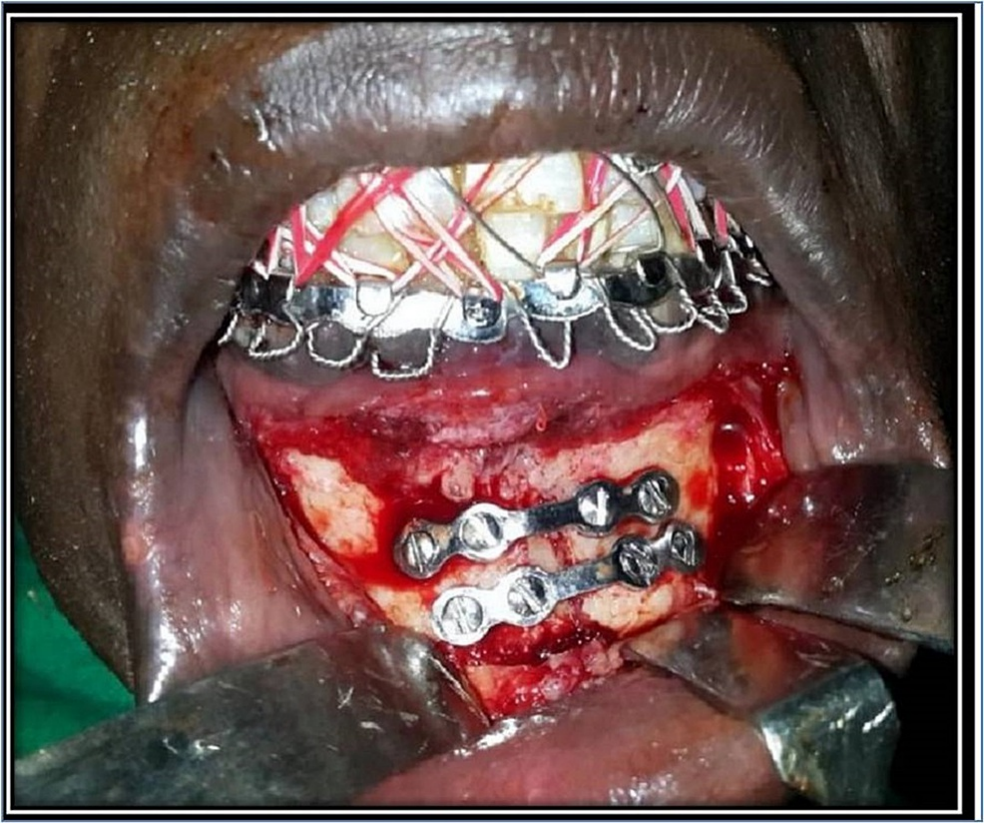
Fixation with two 3D miniplates

In group II, 3D miniplate 2-mm six-hole miniplate with 2.0mm x 10mm mono-cortical stainless steel screws with upper crossbar placed in the subapical position of teeth and injury to dental roots was avoided using mono-cortical screws. In both groups, the plate was adapted and held against the bone with plate holding forceps. A 1.5mm drill bit was used to drill the hole in the outer cortex of the bone perpendicular to the surface of the plate and bone, and screws were fixed and tightened. The fixation of the rest of the screws was achieved similarly (Figure 2).

Irrigation and closure

In case any granulation tissue or foreign bodies around the fractured segment were evident, those were cleaned up. After fixing the plates, the area was thoroughly irrigated with 5% betadine mixed with 0.9% normal saline. The surgical site was then closed in layers. The muscle was secured with 3-0 vicryl interrupted sutures. The mucosa was then closed with 3-0 black silk. An adhesive bandage was applied to the chin extra orally to support the mentalis muscle to prevent drooping of the chin and hematoma formation.

**Figure 3:**
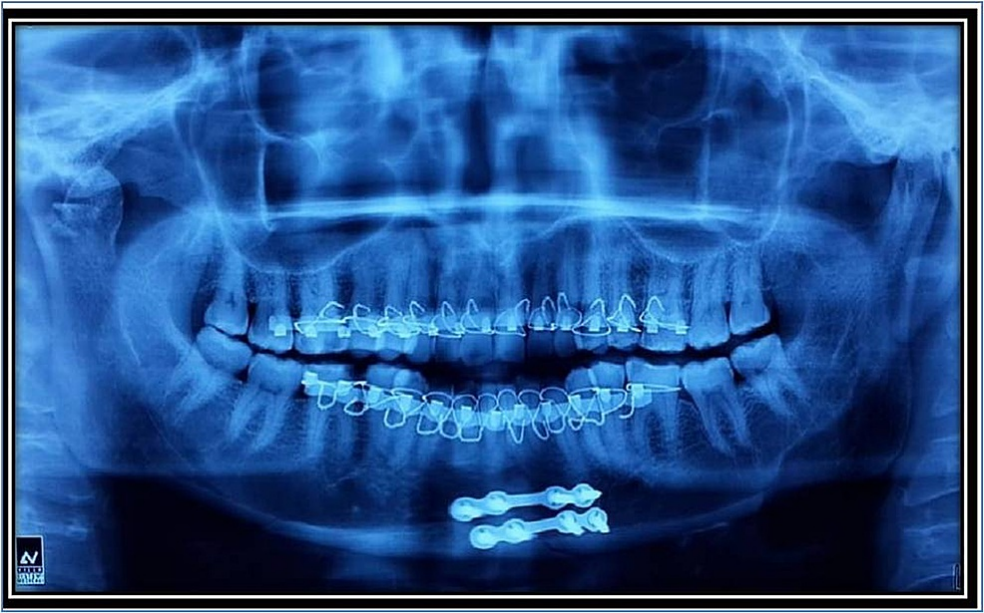
Postoperative orthopantomagram (OPG)

All patients were followed up for three months and assessed clinically and radiographically by taking orthopantomograms (Figure 3). The assessment was made on the immediate postoperative day, third day, fifth day, the seventh day, two weeks, three weeks, four weeks, two months, and three months. 

Statistical analysis

Data analysis was done using IBM Corp. Released 2013. IBM SPSS Statistics for Windows, Version 22.0. Armonk, NY: IBM Corp. The parametric data were analyzed by Student's "t" test while Chi-square/Fisher's test was applied on non-parametric data. Unless otherwise stated, data were presented as mean (standard deviation), and P < 0.05 was considered to be statistically significant. 

## Results

**Figure 4:**
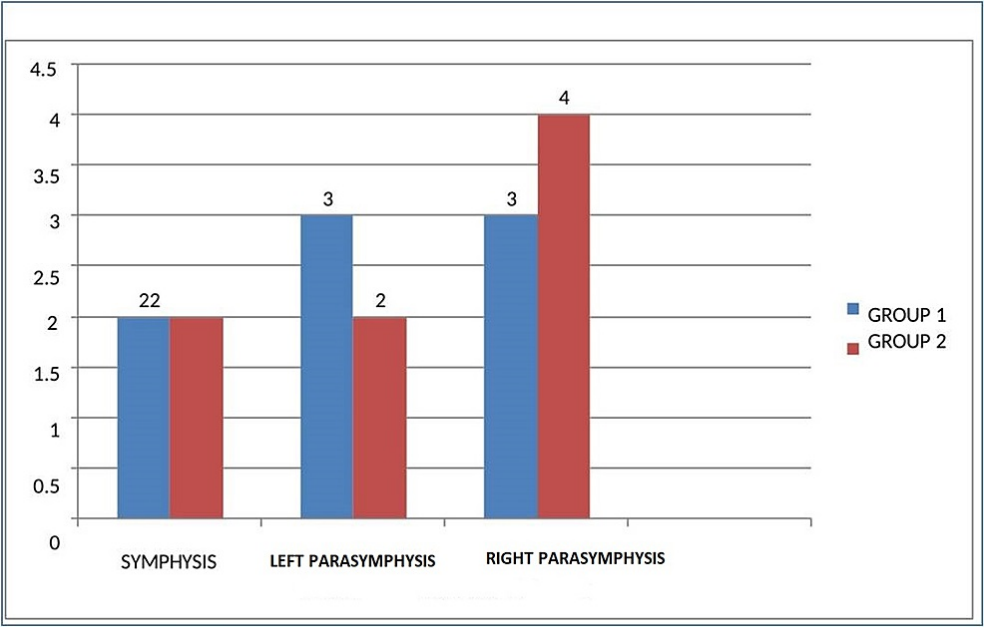
Frequency of fracture site

Out of eight patients in group I, seven were males (87.5%) and one female (12.5%). And out of eight patients in group II, six were males (75%), and two were females (25%). In group I patients, the etiology of injury was road traffic accidents (RTA) in six cases, injury due to interpersonal violence in one case, and injury due to fall from height in one case. And in group II out of eight cases, four cases are due to RTA, two cases are due to interpersonal violence, and the remaining two cases are due to fall from height. Out of 16 cases, 10 patients had parasynthesis fracture, and four cases had symphysis cases (Figure 4). Patients presented to the hospital 1-14 (an average of 5.4 days) days after injury. In all the patients of both groups mild, to moderate occlusal derangement was present.

Time taken for the procedure

Group I: the minimum time was 53 min and the maximum time required was 57 min with a mean of 54.8 min.

Group II: minimum time of 38 min and a maximum of 45 min, and a mean of 40.6 min. Intra-operative time shows statistically significant results, and the values are statistically significant for group II patients when compared to group I patients. The mean ± standard deviation of group I is 54.88 ± 1.642, and group II is 40.62 ± 2.134 (p<0.05) (Table 1).

**Table 1:** Intra-operative time taken for the procedure

Number of Patients	Group I (min)	Group II (min)
1	56	40
2	55	45
3	56	40
4	53	42
5	55	39
6	57	40
7	55	38
8	52	41

Loosening of screws/plates

There was no case reported with loosening of the fracture fragments post-operatively in both groups. Clinically and radiographically, there were no abnormal changes that were detected during the post-operative period.

The discrepancy in the occlusion

The occlusion of the patients was evaluated post-operatively during the first three months to evaluate the progress of the treatment. No patients in either of the groups showed occlusal disturbance (Table 2).

**Table 2:** Frequency distribution of post-operative occlusion p<0.05

Variables			Pre-morbid occlusion		Total
			Achieved	Not achieved	
Group	Group I	Count	8	0	8
		% within group	100	0	100
	Group II	Count	8	0	8
		% within group	100	0	100
Total		Count	16	0	16
		%	100	0	100

Anaesthesia/Paraestheisa of mental nerve

Mild paraesthesia at the soft tissue region supplied by mental nerve was noticed in two patients (25%) of group I, whereas there was no such paraesthesia observed in group II patients. The reason for paraesthesia may be due to manipulation of the mental nerve at the time of surgery adjusting the screws followed by fixation of the bone plates at the fracture site. And this paraesthesia was temporary; it subsided eventually by the fourth week of the postoperative period (Table 3).

**Table 3:** Frequency distribution of post-operative mental nerve paraesthesia

Variables			Mental nerve paraesthesia		Total	Chi-Square value	P value
			Present	Absent			
Group	Group I	Count	2	6	8	2.286	0.467
		% within group	25	75	100		
	Group II	Count	0	8	8		
		% within group	0	100	100		
Total		Count	2	14	16		
		%	12.5	87.5	100		

Evidence of infection/wound dehiscence

Wound dehiscence and infection were noticed in one patient of group I. This can be attributed to inadequate tissue available during suturing, which resulted in tension at the wound closure site and post-operative infection leading to wound breakdown. This patient was kept on antibiotics, advised to maintain good oral hygiene (Table 4).

**Table 4:** Frequency distribution of post-operative wound dehiscence

Variables			Wound Dehiscence		Total		
			Present	Absent			
Group	Group I	Count	1	7	8	1.067	1.00
		% within group	12.5	87.5	100		
	Group II	Count	0	8	8		
		% within group	0	100	100		
Total		Count	1	15	16		
		%	6.25	93.75	100		

Malunion/Non-union

No malunion and non-union were noticed in both groups.

## Discussion

In the present study, out of 16 patients, the major causes for fractures in 10 patients were road traffic accidents (62.5%). These findings were similar to the study conducted by Bormann et al. [6], who found road traffic accidents as a major cause for fractures of the mandible. Depending on the selection criteria, keeping the various anatomical limitations and unique biomechanical behavior of mandibular symphysis and parasymphysis fracture in mind, in this study, conventional miniplates and 3D miniplates for mandibular symphysis and parasymphysis fractures, and the results obtained in this study help in addressing the issue.

In the present study, males are more commonly affected (13 cases, 81%) as compared to females (three cases, 19%). These findings are similar to Haug et al.'s [7] studies. This may be justified by the fact that the males are generally more prone to situations in which there is a higher risk of trauma.

The age group most commonly affected was 21-30 years (56.2%). For occlusion, all the patients were checked preoperatively and postoperatively at various follow-up stages after surgery at the molar/canine region and overjet/overbite relationship. The occlusion of the patients was evaluated post-operatively during the first three months to evaluate the progress of the treatment. No patients in either of the groups showed occlusal disturbance. This coincides with the study of Wittenberg [8], and Guimond [9] report satisfactory occlusion postoperatively in all patients treated with 3D plates.

Meantime taken for miniplate fixation in groups I and II were 54.8 min and 40.6 min, respectively. And the intra-operative time taken was compared based on using a sample T-test, and the results were statistically significant, with group II being with the least mean duration. In group II patients, because of easy adaptability and less screw fixation, the procedure has taken lesser duration when compared to the group I patients. This is not in accordance with Feledy et al.'s study [10].

Mild paraesthesia at the soft tissue region supplied by mental nerve was noticed in two patients (25%) of group I, whereas there was no such paraesthesia observed in group II patients. The reason for paraesthesia may be due to manipulation of the mental nerve at the time of surgery adjusting the screws followed by fixation of the bone plates at the fracture site. And this paraesthesia was temporary; it subsided eventually by the fourth week of the postoperative period. In the present study, out of 16 patients in both groups, two patients in each group had a tooth located in the fracture line. But it did not influence the treatment much. In the present study, in group I, one patient developed an infection at the end of two weeks wound dehiscence and infection were developed. This can be attributed to inadequate tissue available during suturing, which resulted in tension at the wound closure site and post-operative infection leading to wound breakdown. This patient was kept on antibiotics for five days, and daily oral irrigation was done with betadine. The postoperative radiographic assessment showed properly reduced fracture segments with gradual bone healing. No adverse reactions occurred during an entire postoperative period in both groups. Post-operative follow-up was done for three months.

The 3D miniplate group did not show the incidence of wound dehiscence, which is similar to the results obtained by Guimond et al., [9] where the low incidence of wound dehiscence and plate exposure with a 3D plate in comparison to miniplate that might be a result of reduced operating time in 3D miniplate fixation. In the present study, the post-operative radiographic assessment was done for three months, and gradual change in the density was seen in the radiograph, and the overall union was noted within the follow-up period of three months. Kawai et al. conducted the same study [11] to find out the best time to undertake a radiological follow-up examination. They quoted that osteogenic changes were the best radiographic criteria for evaluating follow-up radiographs. The overall union was noted after three months. We also found similar findings.

There are two reasons for the low incidence of complication rates seen with 3D plates: less periosteal stripping and subsequent lesser implant material or foreign material to stabilize the fracture fragments. This was probably why there were 0% complications in group II, but a significant increase in complication rate (30%) in group I [9-11].

Similarly, in none of the cases, postoperative intermaxillary fixation was used. The patients were able to achieve function immediately postoperatively with a reasonable level of success. Though the other areas of the mandible did not pose any problems, the difficulty arose when the fracture line went through the mental foramen or very near to it; in such cases, it was not possible to put the plate unless a nerve translocation procedure was done [12]. However, the limitation of the study is that further studies with larger sample sizes and longer follow-up periods may be required for better evaluation.

## Conclusions

Our experience in using the standard 2D miniplates and 3D miniplates was based on the observations drawn from 16 patients, who could be compared on aspects like age, sex, etiology, and displacement with previous studies. The most common etiology was road traffic accidents followed by assault and fall from height. The procedures were done under local anesthesia. Osteosynthesis was done as per the principles which were designed for the application of the individual systems. Patients were followed up for three months and evaluated for the treatment results and complications. The following inferences could be drawn from the study: the 3D plating system has advantages over conventional 2D miniplates. Quadrangle geometry of plate assures 3D stability of fracture sites as it offers good resistance against torque forces, thereby avoiding the need for inter-maxillary fixation, ensuring early restoration of mandibular function, and reduced rate of infection at fracture site postoperatively. Simplicity, malleability, low profile, ease of application, and reduced infection rate are its advantages over conventional 2D mini plates. It can be concluded that to minimize the rate of postoperative complications for fractures of the symphysis, displaced parasymphysis, and bilateral mandible fractures, 3D plates could be a better option than conventional 2-mm miniplates. However, further studies with larger samples and longer follow-up periods may be required for better evaluation and conclusion.
